# Neuroprotection by the histone deacetylase inhibitor trichostatin A in a model of lipopolysaccharide-sensitised neonatal hypoxic-ischaemic brain injury

**DOI:** 10.1186/1742-2094-9-70

**Published:** 2012-04-18

**Authors:** Bobbi Fleiss, Marie KL Nilsson, Klas Blomgren, Carina Mallard

**Affiliations:** 1 Perinatal Center, Department of Neuroscience and Physiology, Sahlgrenska Academy, University of Gothenburg, Box 432, Gothenburg, 405 30, Sweden; 2 Institute of Neuroscience and Physiology, University of Gothenburg, Box 432, Gothenburg, 405 30, Sweden; 3 Center for Brain Repair and Rehabilitation, Institute of Neuroscience and Physiology, University of Gothenburg, Box 432, Gothenburg, 405 30, Sweden; 4 Karolinska Institutet, Department of Women’s and Children’s Health, Karolinska University Hospital Q2:07, Stockholm, SE 171 76, Sweden; 5 Inserm U676, Hôpital Robert Debré, 48 blvd Serurier, Paris, F-75019, France

**Keywords:** Neonatal, Histone deacetylase, Lipopolysaccharide, Trichostatin A, Hypoxia-ischaemia

## Abstract

**Background:**

Perinatal brain injury is complex and often associated with both inflammation and hypoxia-ischaemia (HI). In adult inflammatory brain injury models, therapies to increase acetylation are efficacious in reducing inflammation and cerebral injury. Our aim in the present study was to examine the neuropathological and functional effects of the histone deacetylase inhibitor (HDACi) trichostatin A (TSA) in a model of neonatal lipopolysaccharide (LPS)-sensitised HI. We hypothesised that, by decreasing inflammation, TSA would improve injury and behavioural outcome. Furthermore, TSA’s effects on oligodendrocyte development, which is acetylation-dependent, were investigated.

**Methods:**

On postnatal day 8 (P8), male and female mice were exposed to LPS together with or without TSA. On P9 (14 hours after LPS), mice were exposed to HI (50 minutes at 10% O_2_). Neuropathology was assessed at 24 hours, 5 days and 27 days post-LPS/HI via immunohistochemistry and/or Western blot analysis for markers of grey matter (microtubule-associated protein 2), white matter (myelin basic protein) and cell death (activated caspase-3). Effects of TSA on LPS or LPS/HI-induced inflammation (cytokines and microglia number) were assessed by Luminex assay and immunohistochemistry. Expression of acetylation-dependent oligodendrocyte maturational corepressors was assessed with quantitative PCR 6 hours after LPS and at 24 hours and 27 days post-LPS/HI. Animal behaviour was monitored with the open-field and trace fear-conditioning paradigms at 25 days post-LPS/HI to identify functional implications of changes in neuropathology associated with TSA treatment.

**Results:**

TSA induced increased Ac-H4 in females only after LPS exposure. Also only in females, TSA reduced grey matter and white matter injury at 5 days post-LPS/HI. Treatment altered animal behaviour in the open field and improved learning in the fear-conditioning test in females compared with LPS/HI-only females at 25 days post-HI. None of the inflammatory mechanisms assessed that are known to mediate neuroprotection by HDACi in adults correlated with improved outcome in TSA-treated neonatal females. Oligodendrocyte maturation was not different between the LPS-only and LPS + TSA-treated mice before or after exposure to HI.

**Conclusions:**

Hyperacetylation with TSA is neuroprotective in the female neonatal mouse following LPS/HI and correlates with improved learning long-term. TSA appears to exert neuroprotection via mechanisms unique to the neonate. Deciphering the effects of age, sex and inflammatory sensitisation in the cerebral response to HDACi is key to furthering the potential of hyperacetylation as a viable neuroprotectant. TSA did not impair oligodendrocyte maturation, which increases the possible clinical relevance of this strategy.

## Background

Perinatal brain injury has a complex aetiology that can involve inflammation in conjunction with hypoxia-ischaemia (HI). Furthermore, experimental studies have shown that inflammation sensitises the neonatal brain to HI injury, possibly by increasing levels of proinflammatory cytokines [1,2]. Brain injury in the newborn causes considerable mortality and long-term neurological sequelae, and treatment and prevention options are limited.

Acetylation of histones is recognised as an important posttranslational modulation of gene expression, including inflammatory genes. Histone deacetylase inhibitor (HDACi) treatment results in an accumulation of acetylated proteins, which has been shown to either increase gene expression by reducing chromatin compaction or reduce gene activation via increases in repressor transcription [3,4]. HDACis reduce expression of proinflammatory-associated molecules such as p53 and NFκB and induce heat shock proteins (HSPs) in sterile adult inflammatory models [5-7]. Also, HDACis decrease lipopolysaccharide (LPS)-induced inflammatory response *in vitro* by reducing inflammatory cell recruitment [8], and they also decrease cytokine expression [9]. Epigenetic regulation, including HDAC class I/II activity, is required for normal brain development, including acquisition of sexually dimorphic brain structure [10] and the proliferation and differentiation of oligodendrocytes [11,12].

Across brain injury models, HDACis categorised by a zinc finger domain, and predominantly inhibiting class I/II HDACs, have been shown to be neuroprotective in adult animals [5,13-15]. As mechanisms of cell death, and thereby the efficacy of neuroprotectants, can differ between adults and neonates [16,17], and because neuroprotectants can disrupt normal developmental processes [18], it is important to investigate potential neuroprotective drugs in immature animals. To date, very little information is available on HDACis as neuroprotectants in immature animals. HDACi treatment following an excitotoxic lesion to the ventral hippocampus in neonatal rats reduced hypersensitivity to apomorphine and deterioration of associative learning [19]. However, researchers in a small neuroprotective study who used valproic acid (VPA) in neonatal rats following HI demonstrated only limited efficacy of VPA and did not examine long-term neuropathological or behavioural follow-up [20]. The possible beneficial effects of HDACis on perinatal inflammation-induced HI brain injury are unknown.

In this study, we used a well-characterised neonatal animal model of LPS-induced HI (LPS/HI) brain injury [1,21,22] to investigate the neuroprotective efficacy of a class I/II HDACi, trichostatin A (TSA). This animal model mimics aspects of brain injury in the human newborn, including the sensitising effects of inflammation to HI injury [23-25]. As sex is now a well-recognised factor in perinatal brain injury mechanisms [26-28], we assessed outcome after LPS/HI and the effects of TSA treatment in males and females separately. Our hypothesis was that TSA would reduce the sensitising effects of LPS on HI brain injury and improve functional outcomes following neonatal LPS/HI via a reduced inflammatory response. We therefore examined TSA effects on white and grey matter injury volume and apoptosis and sought to identify the mechanisms of the neuroprotection by assessing cytokine and chemokine production and microglia activation. To test the hypothesis that neonatal TSA treatment provides long-term beneficial effects, we also assessed brain injury and monitored behavioural outcomes in young adults. Finally, as HDACi activity is critical for oligodendrocyte maturation [12], we sought to determine if there are effects on the program of white matter development following neonatal TSA treatment.

## Methods

### Animals

**Figure 1:**
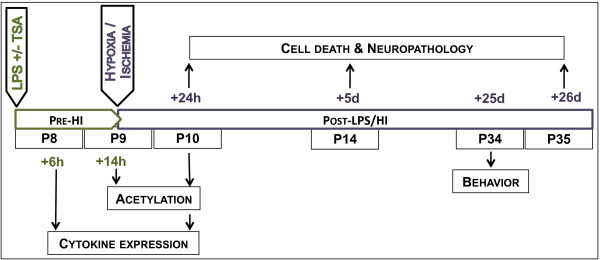
**Schematic representation of the experimental design in the present study.** Analyses of the effects of trichostatin A (TSA) on lipopolysaccharide (LPS)-induced inflammation are in green, and analysis of the neuropathological and behavioural outcomes after LPS/HI with or without TSA are in purple.

C57BL/6 time-mated pregnant mice or dams with postnatal day (P) 7 pups were supplied by Charles River Laboratories International, Sulzfeld, Germany, and maintained at Experimental Biomedicine, University of Gothenburg, Sweden, under specific pathogen-free conditions with a 12-hour light-dark cycle. Standard laboratory chow (B&K, Solna, Sweden) and drinking water were available *ad libitum*. All experiments were approved (No. 374-2009) and conducted within the guidelines of the Gothenburg Animal Ethics Committee. All drugs used were from Sigma-Aldrich (St Louis, MO, USA) unless otherwise stated. The time points of animal treatment and tissue collection are summarised in Figure 1.

### Histone deacetylase dose-response trial

On P8, mice were randomly selected to receive intraperitoneal LPS (O55:B5; 0.3 mg/kg in 0.9% NaCl) and vehicle (10% dimethyl sulphoxide (DMSO) in 0.9% saline) or LPS and either 1, 5 or 10 mg/kg TSA in 10% DMSO (*n* = 8 to 14 per group) or 100 mg/kg VPA in saline (*n* = 14). As VPA at this relatively low dose [5,29] induced death in pups (data not shown), only TSA was used in further studies. A total of 10 litters were used for the TSA dose-response trial, and one male and one female pup were randomly allocated to each treatment group from each litter where possible. Before injection rectal temperature was measured while pups remained in the home cage nest, then they were weighed. At 14 hours after treatment, temperatures and weights were measured again and pups were deeply anaesthetised and perfused intracardially with ice-cold saline, and their brains were removed. The hippocampus and cortex were dissected from the deep grey matter rapidly on ice and were snap-frozen together in liquid nitrogen for subsequent protein analysis.

### Lipopolysaccharide-sensitised brain injury model

At P8, between 18:00 and 21:00 hours, mice were alternately allocated to receive intraperitoneal LPS (O55:B5; 0.3 mg/kg in 0.9% NaCl) and vehicle (10% DMSO in 0.9% NaCl) or LPS and 1 mg/kg TSA (in 10% DMSO). On P9, between 06:00 and 08:00 hours, mice were anaesthetised with isoflurane (5% induction, 1.5% maintenance) in a mixture of nitrous oxide and oxygen (1:1). The left common carotid artery was ligated, and mice were returned to the home cage to recover for 1 hour. The duration of anaesthesia was <5 minutes. After recovery, mice were placed for 50 minutes in a humidified incubator maintained at 36°C perfused with 10.00 ± 0.01% oxygen in nitrogen [1]. We timed the experiment so that hypoxia began at 14 hours after LPS with or without TSA was administered (the evening before at P8), as it is known that this leads to exacerbation of HI injury [1]. Following exposure to hypoxia, pups were returned to the home cage undisturbed for 24 hours (*n* = 7 females per group), 5 days (*n* = 12 males and females per group) or until weaning at P21 and behavioural testing on P35 (*n* = 11 females per group). On P14, animals were deeply anaesthetised and perfused with formalin. Their brains were fixed for a further 24 hours at room temperature and, following embedding in paraffin, sectioned at 5 μm for immunohistochemical assessment of injury. On P10, or at P37 following behavioural testing, animals were deeply anaesthetised, perfused intracardially with saline, and the hippocampus, cortex and subcortical white matter were snap-frozen. Alternatively, at P10 or P37, intracardial perfusion with saline was followed by formalin, and the brains fixed for a further 24 hours at 4°C followed by sucrose cryoprotection and sectioning at 25 μm for immunohistochemical staining.

### Tissue preparation for protein analysis

Frozen brain tissues were homogenised first in PBS using only a handheld homogeniser, and a small aliquot was separately stored for later gene analysis (see below). The remainder was sonicated in ice-cold homogenisation buffer (2 mM ethylenediaminetetraacetic acid and 1% protease inhibitor cocktail (Sigma-Aldrich) in 0.05 M Tris-buffered saline (TBS), and stored at -20°C. The nuclear (P1) fraction was obtained by centrifugation at 800 × *g* for 10 minutes and resuspending the pellet in 0.1 M TBS. Cytosolic proteins were collected by centrifuging the supernatant minus P1 at 9,200 × *g* for 15 min. The supernatant (S2) was decanted and protein concentration for P1 and S2 were determined via a BCA assay.

### Immunoblotting

Immunoblotting was performed as previously described [30]. Samples were mixed with sample buffer and heated at 70°C for 10 minutes before 10 μg of each was loaded and run on a 4% to 12% reducing gel (Invitrogen, Carlsbad, CA, USA), and transferred to nitrocellulose membranes (Bio-Rad Laboratories, Inc, Hercules, CA, USA). Membranes were blocked with TBS-Tween (TBST) buffer (30 mM/L Tris-HCl (pH 7.4), 100 mM/L NaCl and 0.1% Tween) containing 5% fat-free milk powder for 60 minutes at room temperature. After being washed in TBST, membranes were incubated for 60 minutes at room temperature with primary antibodies under the conditions listed in Additional file 1: Table S1. Membranes were washed again, and were incubated with the appropriate peroxidase-labelled secondary antibody (0.25 μg/ml; Vector Laboratories, Burlingame, CA, USA) for 60 minutes. Immunoreactive bands were visualised using the SuperSignal Western Dura substrate (Pierce Biotechnology, Rockford, IL, USA) and a LAS 1000-cooled CCD camera (Fujifilm, Tokyo, Japan) and quantified using Image Gauge software (Fujifilm).

### Cytokine/chemokine assay

Cytokines and chemokines were measured in whole-brain homogenate supernatants from (1) P8 mice killed 6 hours after intraperitoneal treatment with LPS and vehicle (*n* = 15) or LPS and TSA (1 mg/kg; *n* = 16) and (2) P10 mice killed 24 hours after LPS/HI and vehicle (*n* = 10) or LPS/HI and TSA (*n* = 10). Levels of IL-1β, KC/chemokine (C-X-C motif) ligand 1 (CXCL1), monocyte chemotactic protein-1 (MCP*-*1)/chemokine (C-C motif) ligand 2 (CCL2), IL-2, IL-3, IL-4, IL-6, IL-9, IL-10, IL-17, macrophage inflammatory protein 1α (MIP-1α)/CCL3, MIP-1β/CCL4, granulocyte colony-stimulating factor (G-CSF) and TNFα were simultaneously measured using the Bio-Plex Multiplex Cytokine Assay (Bio-Rad Laboratories). The results were normalised to the amount of protein per well as determined using a Bio-Rad DC protein assay.

### Quantitative real-time PCR

A fraction of whole-hemisphere homogenate removed before the addition of homogenisation buffer during preparation for protein analysis was used for RNA extraction. A QIAGEN mini extraction kit (Valencia, CA, USA) was used according to the manufacturer’s instructions, and the quality and concentration of RNA were verified by spectrophotometry. Reverse transcription was performed in duplicate on 500 pg of RNA using a QIAGEN kit as per the manufacturer’s instructions, including DNase treatment. Real-time quantitative PCR was set up using SYBR Green Supermix (Bio-Rad Laboratories) for 40 cycles of a three-step procedure, including 15-second denaturation at 96°C, 30-second annealing at 55°C and a 30-second extension at 72°C. To correlate the threshold cycle to copy number, a standard curve was generated from serial dilutions of a sample with high target gene expression, as ascertained by pilot analysis. A reference gene panel (TATAA Biocenter AB, Göteborg, Sweden) was run on a randomly selected subset of samples from each time point to select the most appropriate reference gene. At 6 and 14 hours after LPS with or without TSA glyceraldehyde 3-phosphate dehydrogenase (*GAPDH*) and hypoxanthine phosphoribosyltransferase 1 (*HPRT1*) expression was used. In samples collected 24 hours after LPS/HI GAPDH was used, and for samples collected 25 days after LPS/HI β-glucuronidase (*GUSB*) was chosen for standardisation. The specific ratio of the gene of interest to the reference gene, or the geometric average of the ratio of the two reference genes was used in analyses.

### Immunohistochemical staining

Immunohistochemistry was performed as described previously for fixed paraffin-embedded [30] and fixed free-floating sections. Briefly, where appropriate, sections were deparaffinised, rehydrated through decreasing concentrations of ethanol and antigen retrieval performed by boiling in citric acid buffer (0.01 M, pH 6.0). Endogenous peroxidase activity was blocked (3% hydrogen peroxide in 0.1 M PBS), as was nonspecific binding (3% serum in 0.1 M PBS), and sections were incubated for 24 to 72 hours with primary antibodies under the conditions listed in Additional file 1: Table S1. Following thorough washing, sections were incubated with the appropriate secondary antibodies (1:250; Vector Laboratories) for 60 minutes. Visualisation of immunoreactivity was achieved using VECTASTAIN ABC Elite reagent with 0.5 mg/ml 3,3′-diaminobenzidine enhanced with 15 mg/ml ammonium nickel sulphate, as well as with 0.01 mg/ml β-glucose oxidase. Sections were dehydrated in graded ethanol and xylene and coverslipped with mounting medium. Sections stained with antiactive caspase-3 were also counterstained with acid fuchsin to visualise pyknotic cells before coverslipping.

### Brain injury evaluation

All evaluations were conducted by an experimenter unaware of the treatment group. Grey and white matter changes were measured in 5-μm-thick serial sections every 375 μm through the brain (n = 5 or 6 levels), stained for microtubule-associated protein 2 (MAP-2) and myelin basic protein (MBP), respectively. Using Micro Image Analysis software (Olympus, Tokyo, Japan) we measured area by manually tracing around the areas of the lateral ventricle, MBP-immunopositive subcortical white matter or the areas of each hemisphere displaying MAP-2 immunopositive staining (uninjured) and immunonegative staining (infarct). The white matter volume, hemispheric volume, lateral ventricle volume, tissue loss and infarct volume were calculated according to the Cavalieri’s principle using the following formula: *V* = *SA* × *P* × *T*, where *V* is total volume, *SA* is the sum of the areas measured, *P* is the inverse of the sampling fraction and *T* is the section thickness, as previously described [1].

### Cell counting

The numbers of ionized calcium-binding adaptor molecule 1 (Iba-1)-positive cells in the whole hemisphere and oligodendrocyte transcription factor 2 (Olig2)-positive cells in the corpus callosum plus the external capsule were determined. All counts were performed by an investigator blinded to treatment group using stereological methods (grid sizes 750 × 750 μm and 100 × 100 μm, respectively) in three to five serial sections spanning approximately bregma -1 to -2.5 (Stereo Investigator version 7; MicroBrightField, Inc, Williston, VT, USA). Numbers of activated caspase-3-and pyknotic-positive cells were assessed in the cortex, hippocampal CA1, dentate gyrus, thalamus and caudate putamen in two or three sections per pup. All cells within a given region were counted and expressed as number of cells per square millimetre.

### Behavioural testing

All testing and training was conducted by an observer blind to the treatment group in a sound attenuated room, under low lighting and during the dark-phase of the circadian cycle. Mice were tested at P35, prior to the onset of regular oestrous [31].

#### Open-field testing

Mice (*n* = 11 per group) were placed into the centre of an unfamiliar 44 × 44-cm dark grey-coloured Plexiglas open-field arena with clean cage bedding (changed between animals) covering the floor, and their behaviour was recorded for 15 minutes with the examiner outside the testing room. Four arenas were run in parallel. Nineteen behavioural variables were extracted from the tracking software (Bioserve Viewer II; Biobserve, St. Augustin, Germany) and are displayed in Table 1. The variables were summarised into 3-minute bins and 15-minute totals for analyses.

**Table 1:** Variables evaluated in the open field experiment and compared within the multivariate analyses

**Total arena**	**Centre zone**^**a**^
Average velocity (cm/second)	Velocity (cm/second)
Track length (cm)	Track length (cm)
Activity (%) (velocity of >0.5 pixels/second or occurrences of head stretches/bobs and tail moves)	Activity (%)
Ambulation (accelerations from stationary to <0.5 pixels/second)	Ambulation
Speed moved in field (0 to 1 cm/minute)	Visits
Speed moved in field (1 to 4 cm/minute)	Visit latencies
Speed moved in field (4 to 10 cm/minute)	Durations
	Head bobs
	Head stretches
	Tail moves
	Number of zone crossings
	Number of rated zone crossings

^a^The centre zone was defined as lying 5 cm or more from the arena edge.

#### Fear conditioning

A schematic of the testing procedure is shown in Additional file 2: Figure S1. On day 1, mice (*n* = 15 to 19 per group) were placed in a 39 × 9.5 × 16.5-cm automatic reflex conditioner box (7530; Ugo Basile Srl, Comerio, Italy) adapted for fear conditioning and baseline freezing scored for 2 minutes. ‘Freezing’ was defined as the cessation of all movement except for that required for breathing, and this was scored every 10 seconds during testing, later adjusted to percentage of time spent freezing. After the mice spent 2 minutes in the testing box, a combined visible and ultraviolet light, and tone (80 db, 670-Hz square sound wave; neutral conditioned stimulus) were presented for 20 seconds, followed 2 seconds later by a scrambled foot shock (0.5 mA; aversive unconditioned stimulus) lasting 2 seconds. Mice were left in the chamber following the foot shock for 30 seconds to allow extinction of any association between the aversive stimulus (foot shock) and the context of the testing apparatus.

The mice were removed from the testing box and returned to their home cages, and the apparatus cleaned with 70% alcohol. Twenty-four hours later the mice were returned to the testing box for 2 minutes and freezing was scored (pretone score) to assess any learned relationship between the testing box and the conditioning stimulus. After this 2-minute pretone period, the tone and light were presented for 30 seconds and freezing was scored in the following 2 minutes (posttone score). Increased posttone freezing relative to pretone score indicated a learned relationship between the conditioning stimulus and the aversive stimulus, thus a type of Pavlovian learning.

### Statistical analysis

Data are presented as means ± SEM. The effects of TSA treatment on outcome measures were assessed for males and females separately. We used a Student’s *t*-test when we compared two groups of normally distributed data and a Mann-Whitney *U* test when data were nonnormal. We used one-way analysis of variance (ANOVA) with a Student’s *t*-test *post hoc* to compare the effects of TSA on groups of three or more. We used two-way ANOVA with a Student’s *t*-test *post hoc* when comparing hemispheres after HI. For ANOVA, TSA treatment was the within-subject variable and hemisphere was the between-subjects variable. *P* < 0.05 was accepted as statistically significant.

Open-field data were analysed using multivariate analyses with principal component analysis followed by partial least squares discriminant analysis (PLS-DA). The results of PLS-DA are presented in an illustrative score plot which can be seen as a projection wherein individuals close to each other in the score plot have similar characteristics. The preprocessing of data consisted of unit variance scaling and mean-centring. The Simca-P + version 11 software program (Umetrics AB, Umeå, Sweden) was used for the calculations. Selected time curves of open-field data based on the output of a loading plot were analysed using two-way ANOVA. In total, 20 mice were run in the open-field experiment. After visual inspection of track files, four (two from each treatment group) had to be discarded because of discontinuous tracking of the animals during recording.

**Figure 2:**
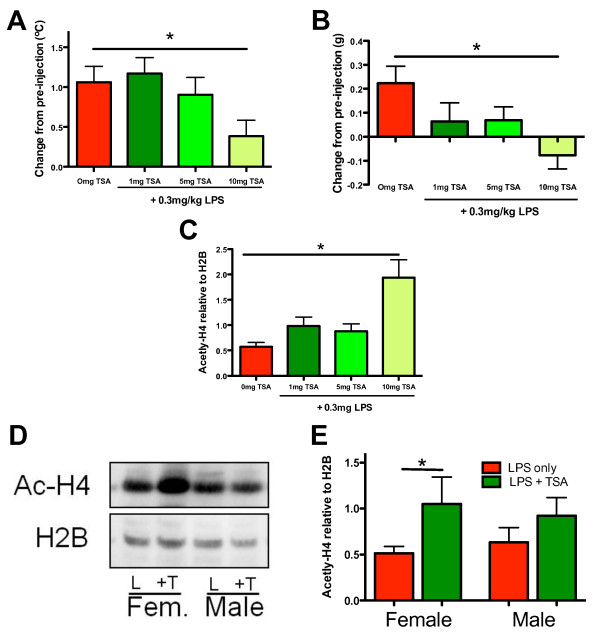
**Trichostatin A (TSA) dose-dependently affects temperature and weight gain and increases histone acetylation in female, but not in male, neonatal mice.**Data shown were gathered at 14 hours after lipopolysaccharide (LPS) treatment with or without TSA. (**A**) Mean rectal temperature (n = 6 to 10 pups). (**B**) Mean change in body weight (n = 6 to 10 pups). (**C**) Mean acetylated histone-4 expression (Ac-H4)normalised to reference protein histone-2B (H2B) (each n = 14). *P <0.05 treatment effect by one-way analysis of variance (ANOVA), #P <0.05 by post hoc t-test. (**D**) and (**E**) Sex-specific Western blot analysis of Ac-H4 (molecular weight 10 kDa) and H2B (molecular weight 15 kDa) showing the effects of LPS (L) and LPS + TSA treatment (+T) in females and males (D) and mean normalised acetylation for female and male mice at 14 hours after intraperitoneal injection (all n = 7). * P<0.05 by Student’s *t*-test.

## Results

### Trichostatin A dose-response trial following lipopolysaccharide exposure

**Figure 3:**
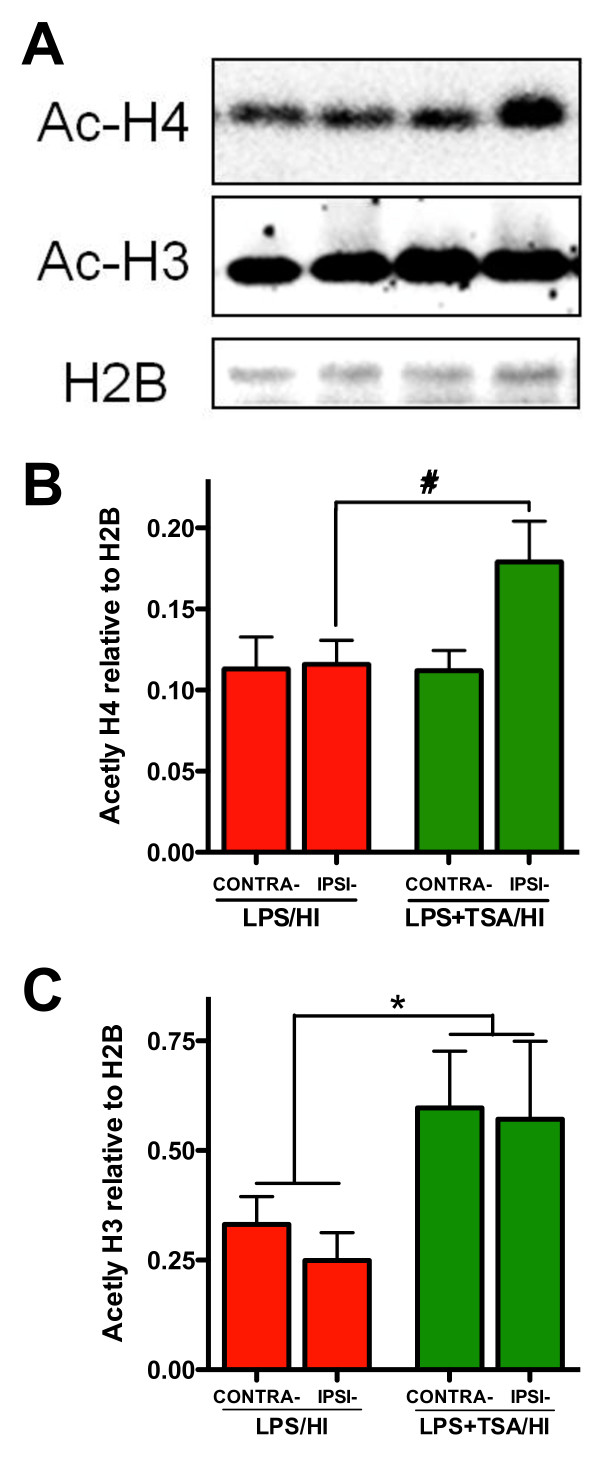
**Trichostatin A (TSA) persistently increases histone acetylation at 24 hours after injury.** (**A**) Western blots of acetylated histone-4(Ac-H4), acetylated histone-3 (Ac-H3, molecular weight 17 kDa) and reference protein H2B in female lipopolysaccharide-sensitised hypoxiaischaemia (LPS/HI) mice (red bars) and LPS + TSA/HI mice (green bars) 24 hours after injury. (**B**) and (**C**) Mean normalised acetylation data are shown for Ac-H4 (B) and Ac-H3 (C) (all n = 7). Data are means ± SEM. *P<0.05 treatment effect by one-way ANOVA, #P<0.05 by post hoc *t*-test.

Using Western blot analysis, we demonstrated that TSA dose-dependently increased H4 acetylation in the brain at 14 hours after LPS exposure (Figure 2) (P < 0.001 by one-way ANOVA), as previously reported [32]. We also monitored indices of pup health, that is, pup weight gain, rectal temperature and the presence of righting reflex. TSA dose-dependently decreased weight gain and rectal temperature (P < 0.05 by one-way ANOVA) (Figures 3A and 3B). Also, pups in the 10-mg group displayed a loss of righting reflex (data not shown), but there was no significant difference in mortality up to 14 hours after treatment ( Additional file 3: Table S2).

*Post hoc* analysis revealed that H4 acetylation was significantly increased by 1 mg/kg TSA (*P* < 0.05 by *t*-test). As this dose caused the least change in weight gain and body temperature and has been shown to be neuroprotective when given prior to an excitotoxic lesion [33], 1 mg/kg was chosen for all further studies.

Additional analysis revealed that the increase in acetylation caused by TSA was significant for female, but not male, pups at 1 mg/kg (Student’s *t*-test, P < 0.05) (Figures 3D and 3E) and 5 mg/kg (data not shown), but that weight changes ( Additional file 3: Table S2) and temperature (data not shown) were not different between the sexes. In this model, exposure of the pups to LPS did not cause hypoacetylation compared to saline-treated pups (data not shown), as reported in models of more severe inflammation [34,35].

### Trichostatin A induces sustained acetylation in the neonatal brain after lipopolysaccharide-sensitised hypoxia-ischaemia

**Figure 4:**
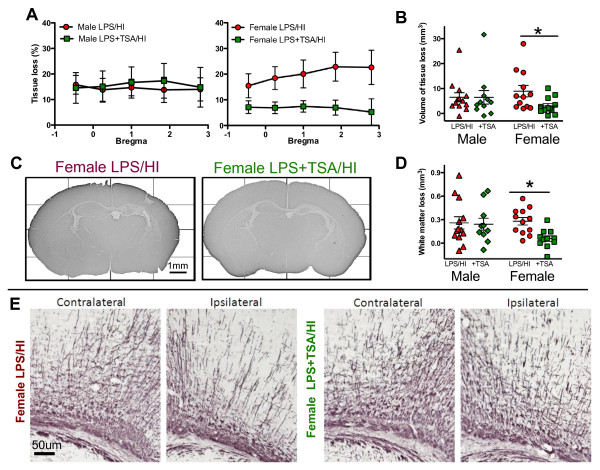
**Trichostatin A (TSA) treatment reduces white and grey matter injury in female, but not male, neonates at 5 days afterlipopolysaccharide-sensitised hypoxia-ischaemia (LPS/HI).** (**A**) Grey matter tissue loss (%) in male and female LPS/HI and LPS + TSA/HI mice at 5 days after HI (all n = 12). (**B**) and (**D**) Total volume of grey matter tissue loss (B) and subcortical white matter loss (D) in male and female LPS/HI and LPS + TSA/HI at 5 days after HI. (**C**) and (**E**) Representative images of microtubule-associated protein 2 (MAP-2)-stained whole hemispheres (C) and myelin basic protein (MBP)-stained subcortical white matter (E) in female LPS/HI and female LPS + TSA/HI mice at 5 days after HI. Data are means ± SEM. *P < 0.05 by Student’s t-test.

Increased acetylation is lost 24 hours after withdrawal of HDACis from cultured oligodendrocytes [11] and 24 hours after HDACi treatment in a middle cerebral artery occlusion model in adults [6]. On the basis of the data derived from the dose-response trial, which showed sex-specific effects of TSA after LPS treatment, we also sought to determine if there were persistent effects of TSA on the epigenome in the female neonatal brain after LPS/HI. At 24 hours after LPS/HI, we observed persistently increased acetylation of histone-3 in both hemispheres of LPS/HI + TSA female mice compared to LPS/HI-only mice (all *n* = 7, *P* < 0.05 by two-way ANOVA) (Figures 3A and 3C). Furthermore, TSA treatment persistently increased histone-4 acetylation at 24 hours after HI in females (all *n* = 7, *P* < 0.05 by two-way ANOVA), an effect localised to the ipsilateral hemisphere (*P* < 0.05 by *post hoc t*-test) (Figure 4A,B).

### Trichostatin A protects grey and white matter at 5-day follow-up but does not reduce acute cell death regulators

Several studies in which adult models of cerebral ischaemia were used have shown neuroprotection following TSA treatment [5,29,35]. To investigate TSA’s effects on short-term neonatal brain injury, tissue loss in cerebral grey and subcortical white matter was assessed 5 days after LPS/HI. Grey and white matter tissue loss was reduced in TSA-treated female pups compared to LPS/HI-exposed females without TSA treatment (both *n* = 12, *P* < 0.05 by Student’s *t*-test) (Figure 4). TSA treatment did not protect the male brain from grey or white matter tissue loss following LPS/HI (both *n* = 12, *P* > 0.05 by Student’s *t*-test). There was no loss of MAP-2 staining in the contralateral hemisphere, indicating lack of injury. The lateral ventricular volume in the ipsilateral hemisphere was increased by approximately 200% compared to the contralateral hemisphere, regardless of TSA treatment and in both sexes (female LPS/HI = 185 ± 29% vs female LPS + TSA/HI = 232 ± 58%, male LPS/HI = 210 ± 16% vs male LPS + TSA/HI = 198 ± 43%; *P* > 0.05 by Student’s *t*-test). Volume of tissue loss in LPS/HI animals without TSA treatment was not different between females and males (females = 8.95 ± 2.29 mm^3^ (*n* = 12), males = 6.48 ± 1.80 mm^3^ (*n* = 12); *P* > 0.05 by Student’s *t*-test). There were no differences in mortality or body weight between groups with or without 1 mg/kg TSA at 5 days after LPS/HI ( Additional file 3: Table S2).

**Figure 5:**
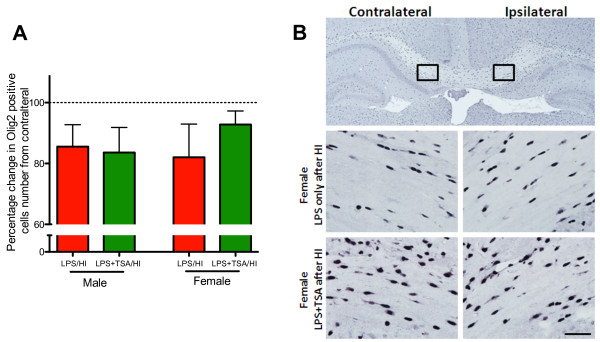
**Trichostatin A (TSA) does not reduce lipopolysaccharide-sensitised, hypoxia-ischaemia (LPS/HI)-induced loss of oligodendrocytes.(A)** Change in contralateral Olig2 number in the subcortical white matter of male and female LPS/HI mice (red bars) and LPS + TSA/HI mice (green bars) at 5 days after HI (all n = 7). **(B)** Representative photomicrographs of Olig2 staining from female LPS/HI and LPS + TSA/HI mice at 5 days after injury. Low-magnification and high-magnification (boxed insets) images are shown. Data are means ± SEM.

Reduced caspase-3 activation and upregulation of heat shock cognate 70 (HSC70) and the LPS-binding protein gelsolin [29,32] have been implicated in neuroprotection by HDACis in adult animals. As such, we also assessed gelsolin expression immediately prior to HI, as well as the number of activated caspase-3-immunopositive cells and protein expression of HSC70 24 hours after LPS/HI. Neither caspase-3-immunopositive cell number nor protein expression of HSP70 or gelsolin was altered by TSA treatment ( Additional file 4: Figure S2; Additional file 5: Table S3). Also, the regional distribution of cell death (as identified by pyknotic cells or caspase-3-positive cells) was not affected by TSA treatment in females at 24 hours after LPS/HI ( Additional file 5: Table S3).

### Trichostatin A treatment did not affect oligodendrocyte number following lipopolysaccharide-sensitised hypoxia-ischaemia

Oligodendrocytes in the neonatal mouse brain are vulnerable to HI injury [36], and we sought to determine whether any loss induced by LPS/HI was prevented by TSA. We counted the number of Olig2-immunopositive cells, a marker of oligodendrocytes, at all stages of development. The number of Olig2-immunopositive cells was decreased by approximately 15% in the ipsilateral compared to the contralateral subcortical white matter in both treatment groups and in both males and females (all *n* = 7, P > 0.05 by Student’s *t*-test) (Figure 5).

**Figure 6:**
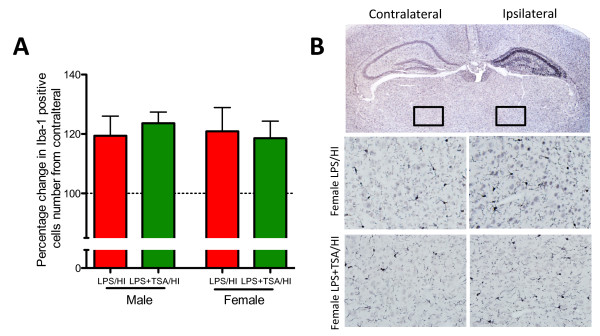
**Trichostatin A (TSA) does not reduce the lipopolysaccharide-sensitised, hypoxia-ischaemia-induced-induced increase of microglia number.**(**A**) Change from contralateral Iba-1 number in the subcortical white matter of male and female LPS/HI mice (red bars) and LPS + TSA/HI mice (green bars) at 5 days after HI (all n = 7). (**B**) Representative photomicrographs of Iba-1 staining from female LPS/HI and LPS + TSA/HI mice at 5 days after injury. Low-magnification and high-magnification (boxed insets) images are shown. Data are means ± SEM.

### Oligodendrocyte maturation/differentiation factors are unaltered by trichostatin A treatment

Normal oligodendrocyte differentiation and maturation rely on HDAC activity to reduce expression of ID2, ID4 and HES5, corepressors of myelin gene transcription [37]. As a preliminary investigation of the safety of TSA treatment in the immature brain, we assessed these HDAC-dependent oligodendrocyte maturational factors and markers of oligodendrocyte maturation (platelet-derived growth factor α (PGDFRα) and MBP). There was no effect of TSA on expression of any of the markers assessed at either 6 hours after LPS with or without TSA treatment ( Additional file 6: Table S4) or at 24 hours or 35 days post-LPS/HI in TSA-treated females ( Additional file 7: Table S5; Additional file 8: Table S6).

### Trichostatin A did not affect lipopolysaccharide-sensitised, hypoxia-ischaemia-induced microglia number

HDACis reportedly confer neuroprotection by causing microglial apoptosis [6,15], and we therefore sought to determine TSA’s effects on microglia number following LPS/HI. Microglia number assessed by Iba-1 staining was increased by approximately 20% in the ipsilateral hemisphere compared to the contralateral hemisphere after LPS/HI. This increase in cell number was localised mainly to the hippocampus but was higher across the entire hemisphere. Microglia number was increased in mice of both sexes, and the increase in the number of microglia was independent of TSA treatment (*n* = 6 to 8, P > 0.05 by Student’s *t*-test) (Figure 5).

**Figure 7:**
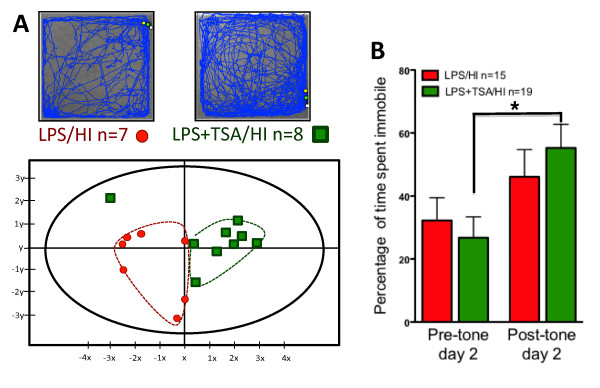
**Neonatal trichostatin A (TSA) treatment altered open-field behaviours and improved learning in young adulthood after lipopolysaccharide-sensitised hypoxia-ischaemia (LPS/HI).**(**A**) Representative trace recordings from LPS/HI- and LPS + TSA/HI-treated mice. (**B**) Output from the multivariate partial least squares discriminant analysis (PLS-DA) of open-field data illustrating significant differences between LPS/HI- and LPS + TSA/HI-treated mice in young adulthood. Each point represents the cumulative value for all behavioural variables for one individual, and red circles represent female LPS/HI. Green squares represent female LPS + TSA/HI. The y-axis is for visualisation purposes only and should not be overinterpreted. The statistics are described further in the Materials and methods section. (**C**) Trace fear-conditioning data illustrating time spent immobile (frozen) pretone and after exposure to the fear-conditioned stimulus (light and tone) on day 2 (n = 15 to 19). Data are means ± SEM. *P<0.05 by post hoc Student’s t-test.

### Cytokine/chemokine production in response to lipopolysaccharide exposure was effected by trichostatin A treatment and sex

Increased proinflammatory cytokine expression is suggested to be one mechanism by which LPS sensitises the neonatal brain to HI injury [1,2]. The ability of HDACis to reduce cytokine production *in vitro* and *in vivo* in adults is suggested to mediate their neuroprotective effects [38,39]. As such, we sought to investigate if there was any effect of TSA on LPS-induced cytokine expression before HI in males or females in this study ( Additional file 9: Figure S3A). Prior to HI in males, TSA treatment increased MIP-1β expression (all *n* = 6 to 8, P < 0.05 by Student’s *t*-test). G-CSF was higher in both males and females treated with TSA compared to those treated with LPS only (*P* < 0.05, n = 6 to 10 by Student’s *t*-test).

Cytokine expression is dependent at least in part on the total expression of, as well as on the ratio of, phosphorylated and nonphosphorylated forms of the NFκB-binding protein IκB [40]. As reduced cytokine expression is observed in conjunction with reduced NFκB activation following HDACi treatment [38], we measured IκB total expression and phosphorylation. In agreement with our observations of only limited effects of TSA treatment on cytokine/chemokine expression, the overall expression of IκB and IκB phosphorylation was unaltered by TSA in males and females (*n* = 7, *P* > 0.05 by Student’s *t*-test) ( Additional file 9: Figures S3B and S3C).

### Trichostatin A treatment modulates cytokine expression after lipopolysaccharide-sensitised hypoxia-ischaemia

The extent of brain injury can be altered by the type and levels of cytokine exposure [41], and as such we investigated TSA’s effects on cytokine expression after LPS/HI in female pups. At 24 hours after LPS/HI, all inflammatory modulators within the range of the assay were increased in the ipsilateral (injured) hemisphere compared to the contralateral (noninjured) hemisphere of both LPS/HI and LPS/HI + TSA pups (all *P* < 0.01 by two-way ANOVA) ( Additional file 10: Figure S4). TSA treatment reduced IL-4 levels in the LPS/HI ipsilateral hemisphere compared with ipsilateral levels following LPS/HI without TSA treatment (*P* < 0.05, by two-way ANOVA). TSA treatment did not affect expression of any of the other cytokines measured in the ipsilateral and contralateral hemispheres.

### Trichostatin A treatment improved learning in early adulthood following neonatal lipopolysaccharide-sensitised hypoxia-ischaemia but did not provide long-term neuroprotection

Several studies, including this one, have found short-term neuroprotective effects of HDACis following cerebral ischaemia. Long-term follow-up, however, including both neuropathological and functional assessment, is lacking. To evaluate long-term TSA effects on neuropathology and behaviour and learning following LPS/HI, we examined brain injury and utilised the open field and trace fear-conditioning paradigms. As locomotor activity and exploratory behaviour and learning in the fear-conditioning test is reduced following neonatal HI [42,43], these behavioural tests were selected to examine long-term effects of TSA following LPS/HI. As TSA, in the short-term part of this study, increased only acetylation and provided neuroprotection in females, studies at P35 were performed in females only.

**Figure 8:**
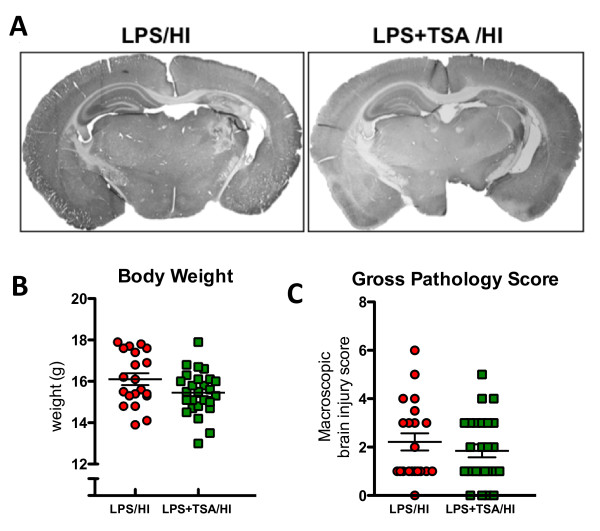
**Neonatal trichostatin A (TSA) treatment altered open-field behaviours and improved learning in young adulthood after lipopolysaccharide-sensitised hypoxia-ischaemia (LPS/HI).(A)** Representative trace recordings from LPS/HI- and LPS + TSA/HI-treated mice. **(B)** Output from the multivariate partial least squares discriminant analysis (PLS-DA) of open-field data illustrating significant differences between LPS/HI- and LPS + TSA/HI-treated mice in young adulthood. Each point represents the cumulative value for all behavioural variables for one individual, and red circles represent female LPS/HI. Green squares represent female LPS + TSA/HI. The *y*-axis is for visualisation purposes only and should not be overinterpreted. The statistics are described further in the Materials and methods section. **(C)** Trace fear-conditioning data illustrating time spent immobile (frozen) pretone and after exposure to the fear-conditioned stimulus (light and tone) on day 2 (*n* = 15 to 19). Data are means ± SEM.^*^P < 0.05 by *post hoc* Student’s *t*-test.

Multivariate analysis was used to analyse the open-field data. A single extreme outlier was identified. As multivariate analyses are sensitive to extreme outliers and to prevent the analysis’s becoming focused on the difference between the one identified outlier and the rest of the group, it was excluded from the final analysis, although this animal’s data is illustrated in the output plot (green point far top left quadrant in Figure 7). The analysis yielded a one-component model, explaining 73% of the variance in the behavioural variables with a predicted validity of 42% (*R*^2^ × (cum) = 0.732; *Q*^2^ (cum) = 0.424)) (Figure 8A). A complementary loading plot for the analysis illustrated behavioural variables as having a variable importance value larger than 1. We created simple time curves and applied two-way ANOVA to these data ( Additional file 11: Figure S5). The simple time curves consolidated the multivariate analysis, and, compared with LPS/HI, only LPS + TSA/HI animals were characterised by spending more time in the centre zone (*P* = 0.016), having a higher occurrence of ambulation in total (*P* = 0.006) as well as in the centre zone (*P* = 0.002), showing longer distance moved in the centre (*P* = 0.006), and demonstrating more head stretches (*P* = 0.039) and tail moves in the centre (*P* = 0.006).

During fear conditioning, female LPS/HI and LPS/HI + TSA adult mice responded identically to the testing apparatus on the day of training, with less than 10% of the time spent immobile before conditioning (light/tone and shock; data not shown). Time spent immobile in response to the training box before conditioning on day 2 was also identical for both groups. Posttone on day 2 two-way ANOVA indicated significantly increased freezing in response to the light and tone, indicative of a learned relationship between these conditioning stimuli and the foot shock (Figure 8B). *Post hoc* analysis indicated that only animals treated with TSA had learnt this association (P = 0.002 by Student’s *t*-test), whereas animals in the LPS/HI (no-TSA) group had not learnt it (P = 0.856 by Student’s *t*-test).

Following behavioural analysis, gross neuropathological score and white matter volume analysis were undertaken. At P35, there was no difference in body weight, injury severity or volume of MBP between LPS/HI only- and LPS/HI + TSA-treated females (Figures 8A and 8B and Additional file 12: Figure S6).

## Discussion

A body of evidence indicates that HDACis are efficacious neuroprotectants across injury models leading to neuroinflammation in adult rodents [44]. Our present study is the first to demonstrate that TSA is neuroprotective in a neonatal LPS-sensitised HI model and specifically that it is protective in a female cohort of animals at any age. TSA at this dose does not affect weight gain or temperature and does not appear to impair the regulatory mechanism governing oligodendrocyte maturation. We also demonstrate that TSA treatment in the neonate can contribute to long-term changes in behaviour following LPS/HI. In the female neonates in this study, the mechanism underpinning neuroprotection by the HDACi TSA was not any of those commonly reported in adult cerebral studies, that is, decrease in cytokine levels, microglial number, caspase-3 or increased HSC70 and gelsolin.

### Trichostatin A is neuroprotective in females only after neonatal lipopolysaccharide-sensitised hypoxia-ischaemia

Our data are in general agreement with those reported previously that HDACis are neuroprotective in models of cerebral injury in adult rodents [5,32,35]. Our data also agrees with a short report of neuroprotection following repetitive VPA treatment, a known HDACi, after HI in neonatal rat [20]. Building from this observation, we have demonstrated efficacy with only a single treatment of HDACi when administered prior to injury. TSA treatment did not protect males from LPS/HI injury in this study or lead to histone hyperacetylation, a key indicator of HDACi activity. Under normal conditions, histone acetylation is higher in male mice than in females from E18 through early postnatal life [45]. Thus a reduced efficacy of TSA compared with females may represent a lower level of available unacetylated lysine residues upon which to act in males. Also, under normal growth conditions, there are regional sex-specific differences in glial density [46], and the immunoreactivity of glia to LPS differs between the sexes [47-49]. These glial effects, together with possible (as yet undescribed) sex differences in the metabolism of TSA and/or blood-brain barrier (BBB) response to LPS or after injury, may have influenced the sex-dependent neuroprotective ability of TSA. Furthermore, sex differences in neonatal stress hormone responsiveness have been described previously [50]. Another possibility is that sex differences in the nuclear receptor of such hormones may have influenced the sex dependent outcome.

Interestingly, despite not significantly altering histone acetylation in males, TSA had a sex-specific effect on LPS-induced cytokine production before HI: higher MIP-1β and G-CSF. These effects likely represent direct or indirect effects of nonhistone HDACi targets that are known to include STAT (signal transducer and activator of transcription), p53, FOXO (Forkhead box O), cell cycle, apoptosis-related genes and RNA processing and stability [51]. These effects may also mediate in part the neuroprotective effects of TSA treatment in females. Also, significant redistribution of epigenetic markers can occur in the absence of changes in total expression [52], and we cannot discount any effect of locus-specific changes in acetylation in males.

### Trichostatin A mediates improved neuropathology later than 24 hours after lipopolysaccharide-sensitised hypoxia-ischaemia injury

TSA treatment failed to reduce cell death 24 hours after LPS/HI, despite improving outcome at 5 days post-HI. This finding is in contrast to reports on reduced cell death in adult HI models at early and late time points postinjury [5,29,32]. Persistent increases in histone-3 and histone-4 acetylation at 24 hours post-LPS/HI suggest that the single dose of TSA is capable of continuing to alter gene expression and improve cell fate at least up to 24 hours post-HI. We were unable to determine any TSA-dependent mechanisms responsible for improvements in neuropathology between 24 hours and 5 days post-HI. Speculatively, the delayed neuroprotection may be mediated in part by acetylation-dependent increases in glial trophic factor production [53], reduced changes in BBB integrity post-HI [54] or increased proliferation and/or neurogenesis [55].

### Neuroprotective mechanisms of action for trichostatin A differ between the adult and neonate

In contrast to adult models of cerebral injury treated with HDACi, improved neuropathogical outcome in the neonate did not correlate with decreased microglial number [6,15] or NFκB-mediated reductions in cytokine expression [38,39,56]. Although we did not investigate microglial apoptosis directly in the present study, we found no TSA-dependent decrease in total microglial number or differences in caspase-3 activity or expression in total cortical extracts from females before or after HI (personal observation, B Fleiss & C Mallard). In agreement with this observation, we have previously been unable to demonstrate a direct relationship between microglial number and neuroprotection following neonatal HI in mice [1,57].

Previous work suggests two reasons why cytokines may not be reduced by HDACi treatment in this LPS-sensitised HI model, as occurs in HI- or inflammation-only models. First, coapplication of TSA and LPS *in vitro* causes bidirectional changes in chemokine and/or cytokine production [15,58,59], and, second, HDACis differentially alter gene expression from activated (LPS-treated) and unstimulated macrophages [8]. To completely exclude that TSA does not improve outcome by altering cytokine expression, however, more extensive temporal analyses post-LPS/HI are necessary.

In addition to cytokine expression and microglia number, we investigated expression of the LPS-binding protein gelsolin and HSC 70, additional mechanisms by which HDACis are reported to mediate neuroprotection in adult cerebral injury models [6,29,32,38,60]. These targets were not altered by TSA treatment in neonatal females, and as such we suggest that they do not mediate neuroprotection in the female LPS-sensitised neonatal brain. This discrepancy in the mechanism of action between previous studies and ours may be related to several factors, including the use of LPS to sensitise the brain to HI. As mentioned above, exposure to LPS in conjunction with HDACis leads to substantial differences in the profile of inflammatory and cell death genes activated compared to LPS or HDACi alone [8]. In addition, neuroprotection with HDACis in adult models is associated with decreased caspase and p53 and increased Akt. LPS sensitisation is associated with an inversion of the neuroprotective changes in these important cell death regulators [5,6,13,29], possibly antagonising the efficacy of TSA in this model. Also possibly modifying the response of the neonate to HDACi treatment is that acetylation of genes, including those affecting cell survival, such as caspase-3 and HSPs, is greater in neonates [59-61]. As such, saturation of acetylation at targets mediating neuroprotection in adults may reduce the ability of TSA to act via these mechanisms in the neonatal brain.

### Long-term behaviours were altered by neonatal trichostatin A treatment prior to lipopolysaccharide-sensitised hypoxia-ischaemia

Functional follow-up in adult rodent models of cerebral injury treated with HDACis consistently correlate with reduced neurological injury severity with improved behavioural outcome [5,13]. However, it has been reported that when using a HDACi to treat lesions of the ventral hippocampus in neonates, there is a discrepancy between neuropathology and behaviour in adulthood [19]. In this study, though injury progressed to the same extent over time, hypersensitivity to apomorphine and deterioration of associative learning, but not anxiety, were reduced by HDACi treatment. Similarly, we found that although TSA did not provide long-term neuroprotection, small changes across multiple indices were observed in the open-field paradigm and learning was improved in the fear-conditioning test. Also, in the present study, we assessed only gross neuropathology in the adult animals. It is possible that these methods were not sensitive enough to identify subtle improvements in neuropathology due to TSA treatment that may underpin altered behaviour in the open field and improved learning.

### Trichostatin A did not effect myelin corepressor expression

Drugs that reduce excitotoxicity and cell death can have deleterious effects on the developing brain [18,62]. A critical concern in considering the safety of HDACis as a neurotherapy is that, during development, decreasing acetylation facilitates maturation of oligodendrocyte precursor cells to myelin-producing oligodendrocytes [12]. In the short or long term after LPS sensitised HI, however, TSA appears to have no effect on the expression of myelin corepressors or the balance of immature to mature oligodendrocytes. Although not conclusive, this finding suggests that the maturation and/or function of Olig2-positive cells are not disrupted by this neuroprotective dosage of TSA.

## Conclusion

This study provides evidence that the HDACi TSA is an efficacious neuroprotectant in females in a neonatal model of LPS-sensitised HI. The sex dependency of TSA as a neuroprotectant is an addition to the accumulating evidence that treatment and patient characteristics need to be more carefully considered and that a one-size-fits-all approach is futile in the search for efficacious neuroprotective strategies [26,63]. Contrary to our original hypothesis, TSA-dependent neuroprotection does not appear to be related to a reduction in LPS- or LPS/HI-induced inflammation. Although we were unable to determine the underlying neuroprotective mechanisms, our study demonstrates that, in neonatal mice, epigenetics can be modified to protect the developing brain without causing gross abnormalities in white matter development. Further studies, including post-injury and repetitive treatment regimens possibly involving additional types of HDACis, will elucidate whether HDACis have a role in future clinically applicable neurotherapies.

## Abbreviations

IκB, Inhibitor of κB; IL, Interleukin; kDa, Kilodalton; NFκB, Nuclear factor κB; PBS, Phosphate-buffered saline; PCR, Polymerase chain reaction.

## Competing interests

The authors declare they have no competing interests.

## Authors’ contributions

CM and BF conceived and designed the experiments. BF performed the experiments. BF, CM and MN analysed and interpreted the data. BF and CM drafted the article. BF, CM, KB and MN revised the article critically for important intellectual content. All authors read and approved the final manuscript.

## Supplementary Material

Additional file **Table S1.**List of antibodies used in the study. Click here for file

Additional file 2 **Figure S1.**Schematic representation of the trace fear conditioning testing procedure. Click here for file

Additional file 3 **Table S2.**Pup characteristics before and after treatment and/or HI. Click here for file

Additional file 4 **Figure S2.**TSA has no effect on the amount of cell death 24 h after LPS sensitized HI or induce HSP-70 expression. **A**) Activated caspase-3 and cresyl violet-stained sections from female LPS + TSA/HI treated mouse, showing injury in areas assessed for levels of cell death (see Table 4). HSP-70 expression; **B**) Western blot of HSC-70 (MW 70 kDa) and reference protein actin (MW 40 kDa) and **C**) mean HSP-70 expression normalized to actin for female LPS/HI and LPS + TSA/HI (all n = 7). Click here for file

Additional file 5 **Table S3.**Cell death in the ipsilateral hemisphere 24 h after LPS sensitized HI in females. Click here for file

Additional file 6 **Table S4.**Oligodendrocyte differentiation/maturation factor expression 6 h after LPS+/− TSA. Click here for file

Additional file 7 **Table S5.**Oligodendrocyte differentiation/maturation factor expression 24 h after LPS sensitized HI in females. Click here for file

Additional file 8: **Table S6.**Oligodendrocyte differentiation/maturation factor expression 35 d after LPS sensitized HI in females. Click here for file

Additional file 9 **Figure S3.**After LPS+/− TSA (before HI) cytokine expression was different dependent on treatment and sex. Cytokine expression adjusted to mg/ml protein per well, LPS only, red; LPS + TSA, green. Mean ± SEM, all n = 6-10 . , Φ, interaction effect and Φ sex effect (P < 0.05) 2 way ANOVA. Click here for file

Additional file 10 **Figure S4.**After LPS sensitized HI cytokine expression was increased in the ipsilateral hemisphere irrespective of TSA treatment. Cytokine expression adjusted to mg/ml protein per well. Mean ± SEM, all n = 6-8. *, P < 0.05 treatment effect in Student’s t-test. Click here for file

Additional file 11 **Figure S5.**Time curves for behavioural variables indicated from the multivariate analysis to have a strong treatment effect. Shown are group mean ± SEM for 3- minute blocks of time, n = 7-8, *, P < 0.05 in a two-way ANOVA. Click here for file

Additional file 12 **Figure S6.**Mean total MBP expression normalized to actin for female LPS/HI (red) and LPS + TSA/HI (green) showing contralateral **(C)** and ipsilateral **(I)** hemispheres (all n = 7), mean ± SEM. Click here for file
