# Supplementary material for: Neuroprotection by the histone deacetylase inhibitor trichostatin A in a model of lipopolysaccharide-sensitised neonatal hypoxic-ischaemic brain injury
Source: J Neuroinflammation. 2012 Apr 18;9:70. doi: 10.1186/1742-2094-9-70 (PMC3420244; doi:10.1186/1742-2094-9-70)
Supplement: Additional file — Table S1.List of antibodies used in the study. [file 1742-2094-9-70-S1.pdf]

Additional File 7.

Supplementary Table 1: List of antibodies used in the study

**Name**	**Product code**	**Application**	**Dilution**	**Source**
MBP; Mouse monoclonal anti-SMI94	SMI-94R	Immunohistochemistry, 5 um paraffin sections	1:10,000	Covance, Inc.
		Western Blotting	1µg/ml	
MAP-2: Mouse monoclonal anti- microtubule-associated protein-2	M4403	Immunohistochemistry, 5 um paraffin sections	1:2000	Sigma-Aldrich
Iba1; Rabbit monoclonal anti-ionized calcium binding adaptor molecule 1	019-19741	Immunohistochemistry, 5 um paraffin sections	1:2000	Wako Chemicals USA
Olig2; Rabbit polyclonal anti-Olig2	AB9610	Immunohistochemistry, 5 um paraffin sections	1:2000	Millipore
Caspase-3; Rabbit monoclonal anti-active caspase-3	559565	Immunohistochemistry, 25 um free floating sections	1:1000	BD Biosciences
Acetyl H4; Rabbit monoclonal anti-acetylated Histone-4	06-698	Western Blotting	1µg/ml	Millipore
Acetyl H3; Rabbit monoclonal anti-acetylated Histone-3	06-588	Western Blotting	1µg/ml	Millipore
Histone 2B; Rabbit monoclonal anti-Histone 2B	07-371	Western Blotting	1µg/ml	Millipore
HSC-70; Mouse monoclonal anti-heat shock cognate 70	Ab90349	Western Blotting	1µg/ml	Abcam
Nrf2; Mouse monoclonal Anti-Nrf2	MAB3925	Western Blotting	5µg/ml	R&D Systems
Actin; Rabbit polyclonal anti-actin	A 2066	Western Blotting	0.1ug/ml	Sigma-Aldrich
