# Supplementary figures and images for: Neuroprotection by the histone deacetylase inhibitor trichostatin A in a model of lipopolysaccharide-sensitised neonatal hypoxic-ischaemic brain injury
Source: J Neuroinflammation. 2012 Apr 18;9:70. doi: 10.1186/1742-2094-9-70 (PMC3420244; doi:10.1186/1742-2094-9-70)

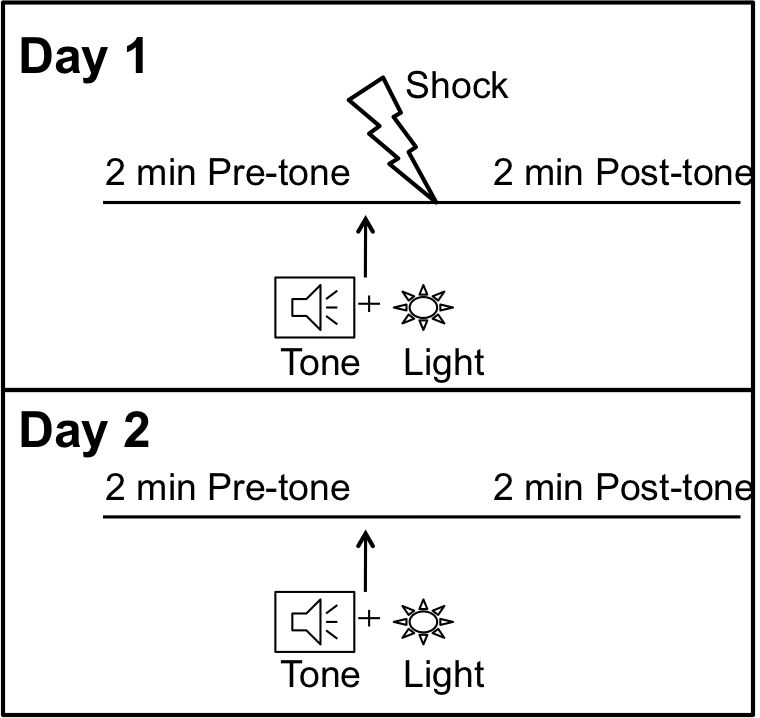

Supplement: Additional file 2 — Figure S1.Schematic representation of the trace fear conditioning testing procedure. [file 1742-2094-9-70-S2.png]

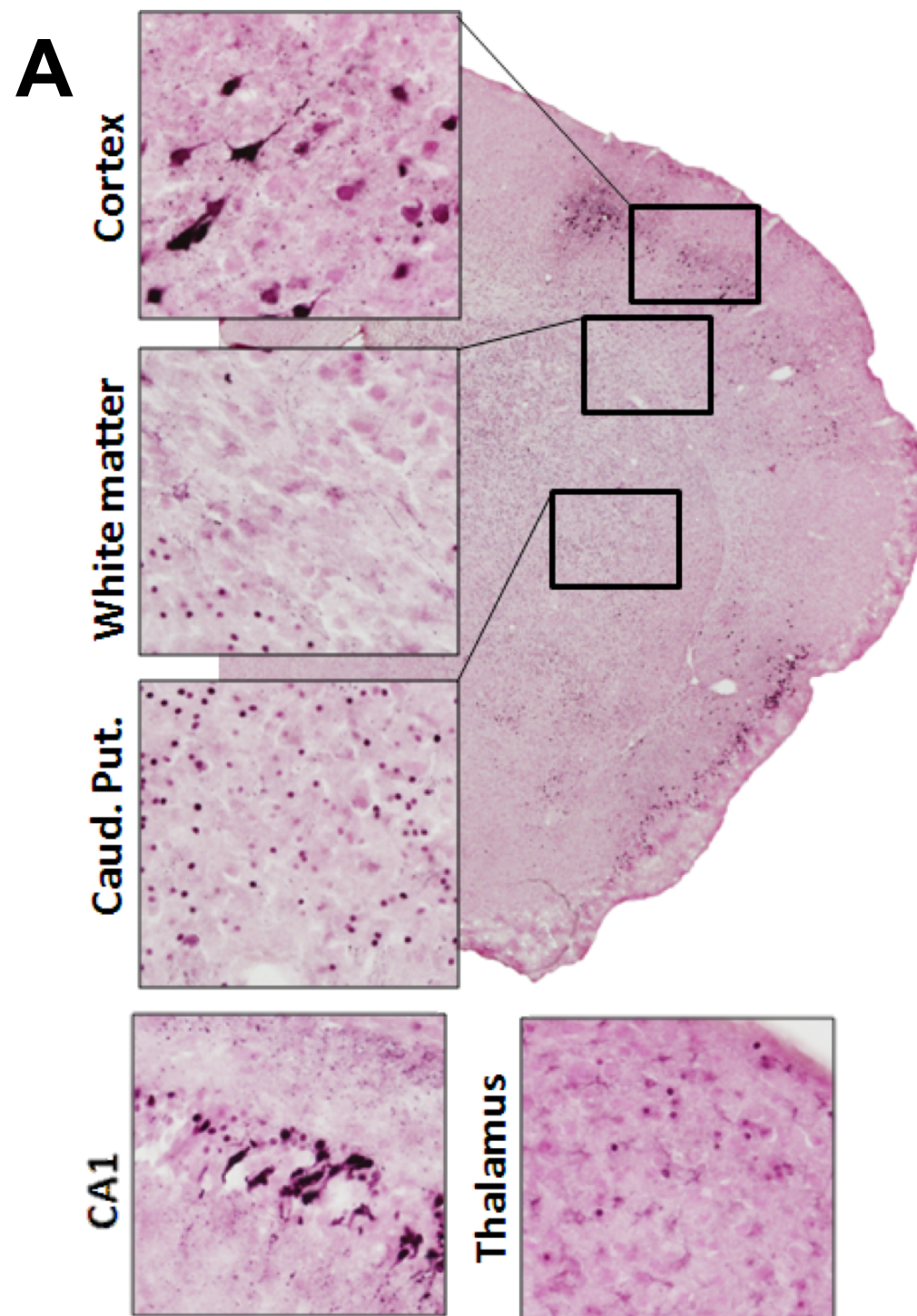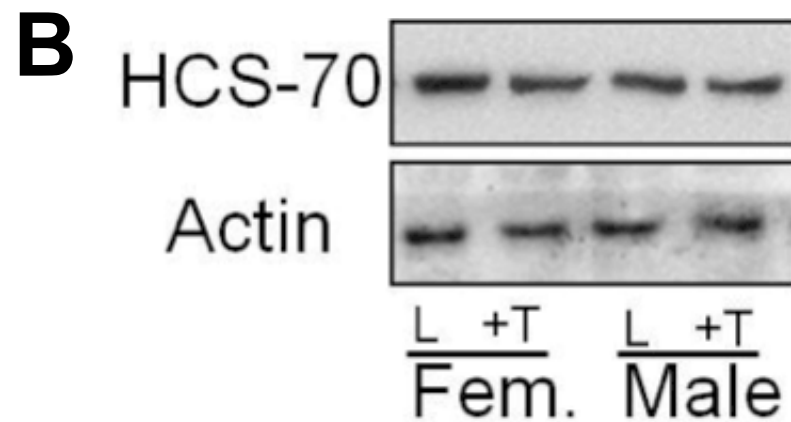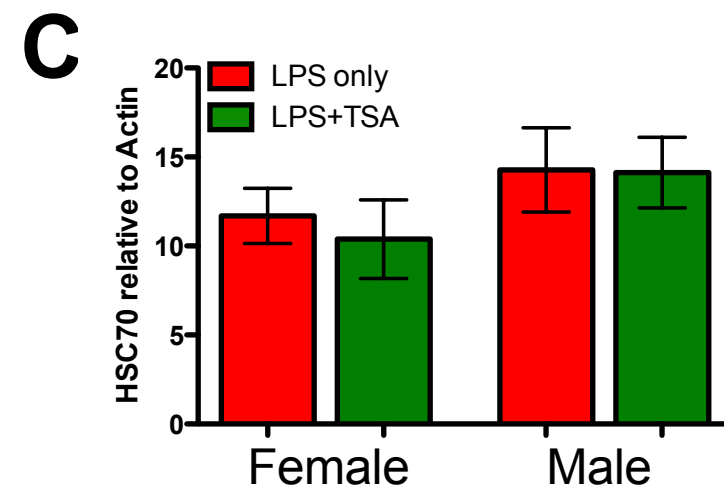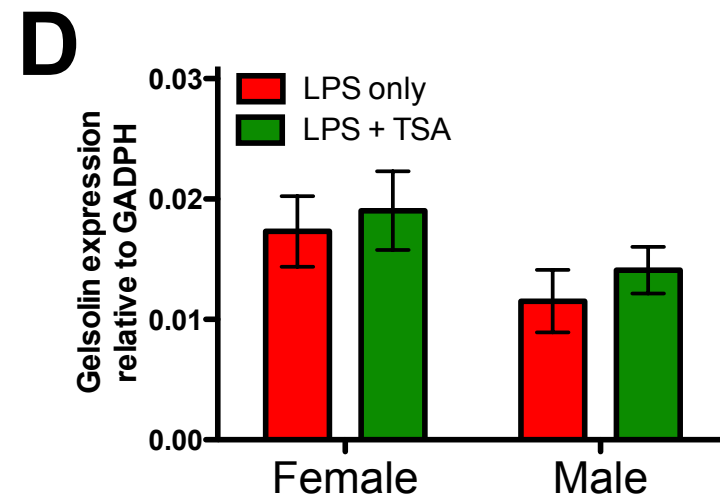

Supplement: Additional file 4 — Figure S2.TSA has no effect on the amount of cell death 24 h after LPS sensitized HI or induce HSP-70 expression. A) Activated caspase-3 and cresyl violet-stained sections from female LPS + TSA/HI treated mouse, showing injury in areas assessed for levels of cell death (see Table 4). HSP-70 expression; B) Western blot of HSC-70 (MW 70 kDa) and reference protein actin (MW 40 kDa) and C) mean HSP-70 expression normalized to actin for female LPS/HI and LPS + TSA/HI (all n = 7). [file 1742-2094-9-70-S4.pdf]

**A**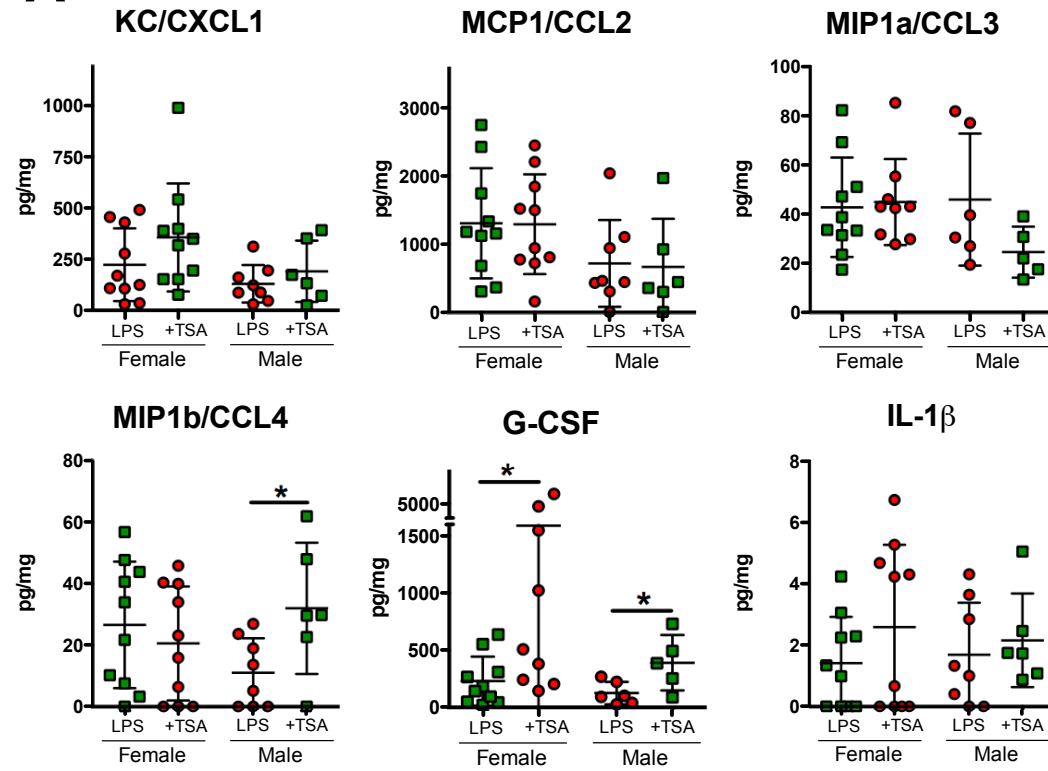**B**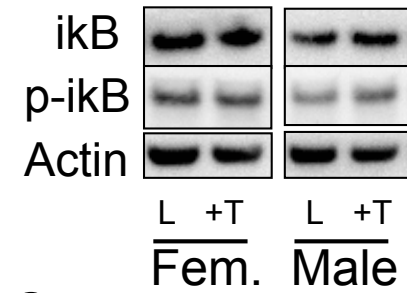**C**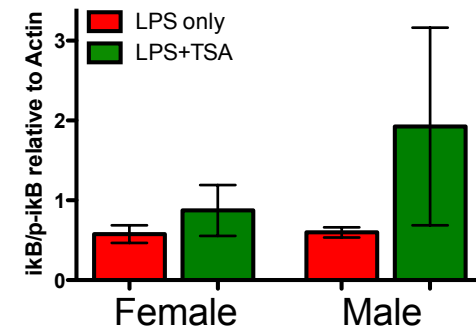

Supplement: Additional file 9 — Figure S3.After LPS+/− TSA (before HI) cytokine expression was different dependent on treatment and sex. Cytokine expression adjusted to mg/ml protein per well, LPS only, red; LPS + TSA, green. Mean ± SEM, all n = 6-10 . , Φ, interaction effect and Φ sex effect (P < 0.05) 2 way ANOVA. [file 1742-2094-9-70-S9.pdf]

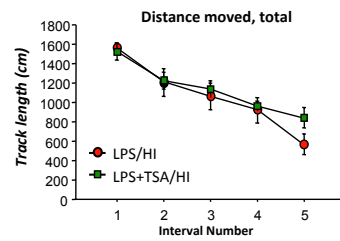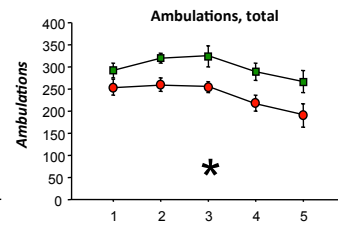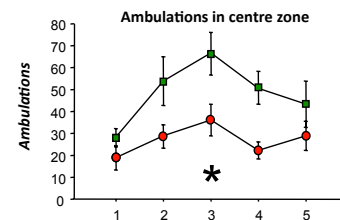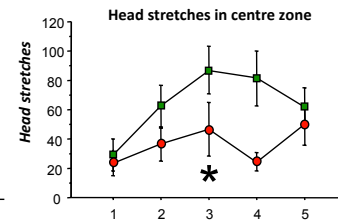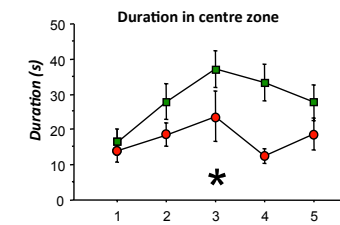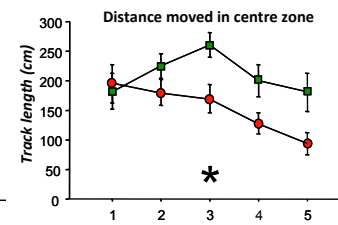

Supplement: Additional file 11 — Figure S5.Time curves for behavioural variables indicated from the multivariate analysis to have a strong treatment effect. Shown are group mean ± SEM for 3- minute blocks of time, n = 7-8, *, P < 0.05 in a two-way ANOVA. [file 1742-2094-9-70-S11.pdf]

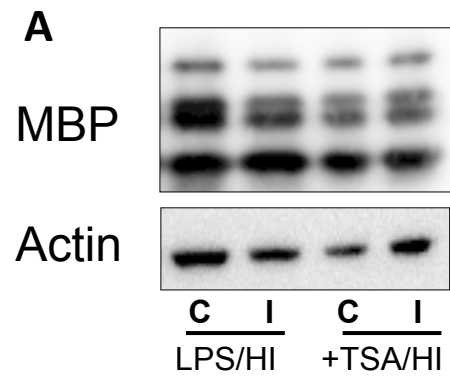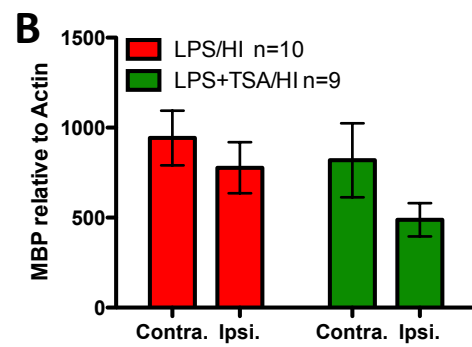

Supplement: Additional file 12 — Figure S6.Mean total MBP expression normalized to actin for female LPS/HI (red) and LPS + TSA/HI (green) showing contralateral (C) and ipsilateral (I) hemispheres (all n = 7), mean ± SEM. [file 1742-2094-9-70-S12.pdf]
