# Supplementary material for: Neuroprotection by the histone deacetylase inhibitor trichostatin A in a model of lipopolysaccharide-sensitised neonatal hypoxic-ischaemic brain injury
Source: J Neuroinflammation. 2012 Apr 18;9:70. doi: 10.1186/1742-2094-9-70 (PMC3420244; doi:10.1186/1742-2094-9-70)
Supplement: Additional file 3 — Table S2.Pup characteristics before and after treatment and/or HI. [file 1742-2094-9-70-S3.pdf]

Additional File 8.

Supplementary Table 2: Pup characteristics before and after treatment and/or HI

	**LPS only**	**LPS + TSA treatment**
Mortality		
Male	18% (3/16)	24% (3/14)
Female	13% (2/15)	29% (5/17)
P8 body weight		
Male	4.82 ± 0.16g	4.91 ± 0.11g
Female	4.60 ± 0.06g	5.00 ± 0.08g
P14 body weight		
Male	6.09 ± 0.45g	5.78 ± 0.46g
Female	5.73 ± 0.31g	5.75 ± 0.37g
P35 body weight		
Female	15.5 ± 0.2g	16.1 ± 0.3g (P=0.06)
