# Supplementary material for: Neuroprotection by the histone deacetylase inhibitor trichostatin A in a model of lipopolysaccharide-sensitised neonatal hypoxic-ischaemic brain injury
Source: J Neuroinflammation. 2012 Apr 18;9:70. doi: 10.1186/1742-2094-9-70 (PMC3420244; doi:10.1186/1742-2094-9-70)
Supplement: Additional file 5 — Table S3.Cell death in the ipsilateral hemisphere 24 h after LPS sensitized HI in females. [file 1742-2094-9-70-S5.pdf]

Additional File 9.

Supplementary Table 3. Cell death in the ipsilateral hemisphere 24 h after LPS sensitized HI in females

		LPS/HI	LPS+TSA/HI
Cortex	Caspase-3 +ve	4.11 ± 2.10	2.92 ± 0.84
	Pyknosis +ve	0.15 ± 0.06	0.12 ± 0.03
Subcortical White Matter	Caspase-3 +ve	0.75 ± 0.36	0.83 ± 0.25
	Pyknosis +ve	0.15 ± 0.07	0.13 ± 0.05
CA1	Caspase-3 +ve	1.39 ± 0.27	3.03 ± 1.41
	Pyknosis +ve	na	0.05 ± 0.04
Dentate Gyrus	Caspase-3 +ve	0.06 ± 0.05	na
	Pyknosis +ve	1.70 ± 0.33	3.25 ± 0.72
Thalamus	Caspase-3 +ve	0.57 ± 0.14	0.58 ± 0.14
	Pyknosis +ve	2.80 ± 0.47	2.48 ± 0.82
Caudate Putamen	Caspase-3 +ve	0.65 ± 0.05	0.50 ± 0.19
	Pyknosis +ve	4.41 ± 2.45	5.91 ± 2.72

All values are cells per mm^2 and na=no cells counted of that type.
