# Supplementary material for: Neuroprotection by the histone deacetylase inhibitor trichostatin A in a model of lipopolysaccharide-sensitised neonatal hypoxic-ischaemic brain injury
Source: J Neuroinflammation. 2012 Apr 18;9:70. doi: 10.1186/1742-2094-9-70 (PMC3420244; doi:10.1186/1742-2094-9-70)
Supplement: Additional file 6 — Table S4.Oligodendrocyte differentiation/maturation factor expression 6 h after LPS+/− TSA. [file 1742-2094-9-70-S6.pdf]

Additional File 10.

Supplementary Table 4. Oligodendrocyte differentiation/maturation factor expression 6 h after LPS+/- TSA

		**LPS only**	**LPS + TSA**	
ID2	*Female*	4.579 ± 1.078	4.194 ± 0.601	*
	Male	2.872 ± 0.524	2.996 ± 0.398	
ID4	*Female*	35.46 ± 6.796	25.64 ± 4.314	*
	Male	22.96 ± 3.203	18.69 ± 3.452	
HES5	*Female*	0.153 ± 0.054	0.137 ± 0.034	*
	Male	0.082 ± 0.010	0.067 ± 0.006	
Olig2	*Female*	0.288 ± 0.082	0.277 ± 0.061	
	Male	0.233 ± 0.057	0.190 ± 0.048	
PDGFRα	*Female*	0.034 ± 0.007	0.033 ± 0.006	
	Male	0.025 ± 0.005	0.022 ± 0.004	
MBP	*Female*	1.380 ± 0.258	1.336 ± 0.686	
	Male	0.713 ± 0.192	1.152 ± 0.281	

Gene expression expressed as the geometric mean of data normalized to GAPDH and HPRT1. LPS only female n=9, male n=9; LPS+TSA female n=9, male n=10. *, $P<0.05$ comparing male and female at the same time point; 2-way ANOVA.
