# Supplementary material for: Neuroprotection by the histone deacetylase inhibitor trichostatin A in a model of lipopolysaccharide-sensitised neonatal hypoxic-ischaemic brain injury
Source: J Neuroinflammation. 2012 Apr 18;9:70. doi: 10.1186/1742-2094-9-70 (PMC3420244; doi:10.1186/1742-2094-9-70)
Supplement: Additional file 7 — Table S5.Oligodendrocyte differentiation/maturation factor expression 24 h after LPS sensitized HI in females. [file 1742-2094-9-70-S7.pdf]

Additional File 11.

Supplementary Table 5. Oligodendrocyte differentiation/maturation factor expression 24 h after LPS sensitized HI in females

		LPS/HI only	LPS + TSA/HI
ID2	*Contralateral*	0.084 ± 0.013	0.076 ± 0.013
	Ipsilateral	0.080 ± 0.013	0.887 ± 0.172
ID4	*Contralateral*	0.735± 0.615	0.717 ± 0.120
	Ipsilateral	0.615 ± 0.104	0.734 ± 0.138
HES5	*Contralateral*	0.006 ± 0.002	0.005 ± 0.002
	Ipsilateral	0.007 ± 0.002	0.005 ± 0.002
Olig2	*Contralateral*	0.025 ± 0.002	0.023 ± 0.003
	Ipsilateral	0.026 ± 0.007	0.027 ± 0.007
PDGFRα	*Contralateral*	0.110 ± 0.030	0.007 ± 0.023
	Ipsilateral	0.072 ± 0.020	0.051 ± 0.016
MBP	*Contralateral*	0.067 ± 0.011	0.059 ± 0.007
	Ipsilateral	0.063 ± 0.011	0.056 ± 0.006

Gene expression expressed as data normalized to GAPDH. LPS/HI female n=7; LPS+TSA/HI female n=9.
