# Supplementary material for: Neuroprotection by the histone deacetylase inhibitor trichostatin A in a model of lipopolysaccharide-sensitised neonatal hypoxic-ischaemic brain injury
Source: J Neuroinflammation. 2012 Apr 18;9:70. doi: 10.1186/1742-2094-9-70 (PMC3420244; doi:10.1186/1742-2094-9-70)
Supplement: Additional file 8: — Table S6.Oligodendrocyte differentiation/maturation factor expression 35 d after LPS sensitized HI in females. [file 1742-2094-9-70-S8.pdf]

Additional File 12.

Supplementary Table 6. Oligodendrocyte differentiation/maturation factor expression 35 d after LPS sensitized HI in females

		LPS/HI only	LPS + TSA/HI
ID2	*Contralateral*	68.00 ± 4.90	75.76 ± 8.39
	Ipsilateral	66.38 ± 6.42	53.22 ± 5.79
ID4	*Contralateral*	1046 ± 99.64	1071 ± 155.10
	Ipsilateral	1076 ± 157.00	931.0 ± 99.29
HES5	*Contralateral*	1.731 ± 0.179	1.996 ± 0.283
	Ipsilateral	1.244 ± 0.157	1.312 ± 0.194
Olig2	*Contralateral*	0.774 ± 0.135	0.688 ± 0.179
	Ipsilateral	0.677 ± 0.148	0.582 ± 0.189
PDGFRα	*Contralateral*	0.195 ± 0.021	0.290 ± 0.081
	Ipsilateral	0.157 ± 0.024	0.268 ± 0.090
MBP	*Contralateral*	942.5 ± 152.0	777.4 ± 141.4
	Ipsilateral	819.0 ± 205.3	487.9 ± 92.65

Gene expression expressed as data normalized to the arithmetic mean of GUSB and GAPDH. n=6-12
